# Delta Sleep-Inducing Peptide Recovers Motor Function in SD Rats after Focal Stroke

**DOI:** 10.3390/molecules26175173

**Published:** 2021-08-26

**Authors:** Elena A. Tukhovskaya, Alina M. Ismailova, Elvira R. Shaykhutdinova, Gulsara A. Slashcheva, Igor A. Prudchenko, Inessa I. Mikhaleva, Oksana N. Khokhlova, Arkady N. Murashev, Vadim T. Ivanov

**Affiliations:** 1 Biological Testing Laboratory, Branch of Shemyakin and Ovchinnikov, Institute of Bioorganic Chemistry, Russian Academy of Sciences, Pushchino, Prospekt Nauki, 6, 142290 Moscow, Russia; ismailowa.a.m@yandex.ru (A.M.I.); shaykhutdinova@bibch.ru (E.R.S.); slashcheva_ga@mail.ru (G.A.S.); khohlova@bibch.ru (O.N.K.); murashev@bibch.ru (A.N.M.); 2 Laboratory of Peptide Chemistry, Shemyakin and Ovchinnikov Institute of Bioorganic Chemistry, Russian Academy of Sciences, Miklukho-Maklaya Street, 16/10, 117997 Moscow, Russia; iaprud@ibch.ru (I.A.P.); inessamikh@rambler.ru (I.I.M.); ivavt37@gmail.com (V.T.I.)

**Keywords:** rats, stroke, motor function, DSIP

## Abstract

*Background and Objectives*: Mutual effect of the preliminary and therapeutic intranasal treatment of SD rats with DSIP (8 days) on the outcome of focal stroke, induced with intraluminal middle cerebral occlusion (MCAO), was investigated. *Materials and Methods*: The groups were the following: MCAO + vehicle, MCAO + DSIP, and SHAM-operated. DSIP or vehicle was applied nasally 60 (±15) minutes prior to the occlusion and for 7 days after reperfusion at dose 120 µg/kg. The battery of behavioral tests was performed on 1, 3, 7, 14, and 21 days after MCAO. Motor coordination and balance and bilateral asymmetry were tested. At the end of the study, animals were euthanized, and their brains were perfused, serial cryoslices were made, and infarction volume in them was calculated. *Results*: Although brain infarction in DSIP-treated animals was smaller than in vehicle-treated animals, the difference was not significant. However, motor performance in the rotarod test significantly recovered in DSIP-treated animals. *Conclusions*: Intranasal administration of DSIP in the course of 8 days leads to accelerated recovery of motor functions.

## 1. Introduction

Delta sleep-inducing peptide (DSIP) is a multifunctional regulatory peptide. It was first isolated from cerebral venous blood of rabbits after low-frequency (‘hypnogenic’) electrical stimulation of the intralaminar thalamic nuclei by the Schoenenberger–Monnier group from Basel in 1977 [1]. Subsequently, the functions of the DSIP were actively investigated. Consequently, in the work of Iyer et al. (1988), the effect of DSIP on the induction of slow-wave sleep and sleep-related growth hormone release was shown [2]. In a later study, the absence of the effect of DSIP in the formation of sleep was shown when injected into the nucleus raphe dorsalis of rats [3]. A wide range of studies conducted to identify the biological activities of DSIP showed that endogenous DSIP or DSIP-like peptides have regulatory activity and play an important role in endocrine regulation. Thus, it has been shown that DSIP reduces the basal level of corticotropin [4,5,6,7,8,9,10,11,12,13,14] and stimulates the secretion of luteinizing hormone and the release of somatoliberin and somatotropin [8]. DSIP has a wide range of physiological activities, some of which are not related to each other [15]. It has stress-protective and adaptive activity [16,17,18,19,20]. DSIP acts as an adaptogen at amphetamine-induced stereotypy, which is a model of schizophrenia-like condition in humans. It normalizes brain metabolism injured with long-term amphetamine treatment. It is also shown that DSIP normalizes MAO activities through serotonin adrenergic systems [21]. Konorova et al. elucidated the ability of DSIP (as the medical product deltaran) to reduce the lethality of stress in low-resistant rats [22]. DSIP also has neuroprotective properties. Accordingly, the treatment of Wistar rats with DSIP several hours prior to global brain ischemia improved locomotor functions and reduced lethality [23]. DSIP reduced neuronal activity and improved blood supply of the brain of stressed animals subjected to brain ischemia [24]. There are several reports suggesting that DSIP reduces stress-induced overproduction of free radicals in CNS and prevents neuronal death [16,25]. Investigation of DSIP at a model of carotid arteries occlusion in different age groups of male rats revealed DSIP’s ability to correct the impaired balance of brain neuromediators hypoxia [25]. DSIP has also been shown to potentiate the action of the anticonvulsant drug Valproate in metaphit-provoked generalized epilepsy induced by audiogenic stimulation in adult rats, indicating the potential for using DSIP in combination with antiepileptic drugs for the treatment of seizures [26].

The aim of the present study was to investigate the effect of mutual preventive and therapeutic intranasal treatment of SD rats with DSIP on the consequences of focal stroke.

## 2. Materials and Methods

### 2.1. Animals

Adult male Sprague-Dawley (SD) rats of 320–380 g were used. All animals were SPF and were obtained from the Pushchino Nursery for Laboratory Animals (Pushchino, Russia). All procedures and manipulations with animals were approved by the Committee for Control over Care and Use of Laboratory Animals of BIBCh RAS and were carried out in accordance with the EU Directive 2010/63/EU for animal experiments and Animal Control and Use Committee (IACUC) protocol number 683/19. The Biological Testing Laboratory, Branch of Shemyakin, and the Ovchinnikov Institute of Bioorganic Chemistry, Pushchino, Moscow Region, Russia, where the experiments were followed, has AAALAC accreditation (can be found as Russian Academy of Sciences Biological Testing Laboratory-Branch of Shemyakin & Ovchinnikov Institute of Bioorganic Chemistry Pushchino, Moscow Region, Russia by clicking on the link https://www.aaalac.org/accreditation-program/directory/directory-of-accredited-organizations-search-result/#R, (accessed on 24 August 2021). The rats were maintained on 12 h light/dark cycle with unlimited access to food and water.

### 2.2. Drugs

DSIP (H-Trp-Ala-Gly-Gly-Asp-Ala-SerGly-Glu-OH) was synthesized in the Laboratory of Peptide Chemistry, Shemyakin, and the Ovchinnikov Institute of Bioorganic Chemistry, Russian Academy of Sciences, Moscow, Russia, as described elsewhere [26].

### 2.3. Middle Cerebral Artery Occlusion Procedure (MCAO)

A total of 26 mature male SD rats were used. Animals were anesthetized intramuscularly with a ketamine–xylazine mixture (30 mg/kg ketamine and 12 mg/kg xylazine). The total injection volume was 1.2 mL/kg. The right temporal muscle was dissected, temporal bone was dried with cotton balls, and a laser Doppler flow (LDF) probe (PeriFlux 5001, PR407-1 Small Straight Probe) was attached to the temporal bone to measure regional cerebrovascular blood flow (rCBF). A midline cervical incision was made to expose the right common carotid artery (CCA). Loose ligatures were placed on CCA and internal carotid artery (ICA), followed by two loose ligatures on the external carotid artery (ECA) at a point 2–3 mm distal from the bifurcation of CCA. The occipital artery was ligated permanently and the pterygopalatine artery (PPA) was ligated for the MCAO procedure duration. Occluding sutures were prepared using a silicon–Teflon monofilament of 0.13 mm diameter (STROFT GTM, Germany) with a heat blunted end (0.3 mm diameter) coated with 0.5% (*w*/*v*) poly-l-lysine solution and dried under a heating lamp for 1 h [27]. The intraluminal suture was inserted through a small puncture opening in ECA and advanced into the ICA to approximately 18 mm from the bifurcation (depending on a rat weight) until a slight resistance occurred. When decreased blood flow was observed, the occlusion suture was left in place for 90 min. Ischemia was confirmed by a more than 70% rCBF decrease compared with the baseline value. After 90 min of occlusion, the suture was withdrawn. Blood flow restitution to 90–120% of baseline in 10 min was recognized as a successful reperfusion. The ligatures were removed from the CCA, ICA, and PPA, while the ECA was permanently ligated above and below the opening. The LDF probe was detached, and the wounds were closed with a twisted suture. Sham-operated rats were subjected to the same surgical procedure except for the MCA occlusion. The rectal temperature was maintained at 37 °C using a heating pad until the animals recovered from anesthesia. After surgery, the rats were returned to their cages and allowed free access to food and water.

### 2.4. Groups and Dosing Regimen

The animals were divided into three groups: animals subjected to MCAO and treated with vehicle—MCAO + vehicle (saline) (*n* = 9); animals subjected to MCAO and treated with DSIP—MCAO + DSIP (*n* = 10); sham-operated animals—SHAM (*n* = 7). The animals received DSIP nasally 60 (±15) minutes prior to the occlusion and for 7 days after reperfusion. The DSIP dosage was 120 µg/kg (volume of treatment was 100 µL/kg).

### 2.5. Behavioral Testing

Each rat was subjected to a series of behavioral tests on days 1, 3, 5, 7, 14, and 21 after a stroke. Motor deficits of balance and coordination were assessed by rotarod testing. Drug-induced rotations asymmetry was tested. All behavioral testing was performed during the animals’ light cycle.

#### 2.5.1. Rotarod Test

Quantitative assessment of motor coordination and balance was performed using an automated rotarod (Dual Species Economex Rota-Rod, Columbus Instruments). All animals were trained on the rotarod before the MCAO procedure for 3 consecutive days. This training consisted of three trials per day on each of these 3 days (nine trials in total). Each trial consisted of placing the animal onto the rotarod, which was revolving at a constant speed (5 rpm/min), and then the rotarod was switched to acceleration mode (0.6 rpm/min). Fall latency was defined as the time during which the animal balanced on the rotating rod from the moment the acceleration was switched on until the moment it fell off the rod. The countdown started from point 0 when acceleration was turned on and continued until the animal fell or was held on a rotating rod (squeezing its paws) for two full revolutions (720°). According to the results of training and preliminary testing, the animals were selected into groups so that in all groups there were animals with homogeneous results. The purpose of this pre-testing was to identify groups of animals with similar performance in the rotarod test in order to more accurately determine how MCAO and MCAO + DSIP treatments might affect this performance. Starting from the first day after MCAO, consecutive sessions of rotarod testing were started. In each daily session, testing was repeated three times with 5 min intervals between each trial. Animals unable to grasp the rotating rod were given a latency value of 0 s. Latency times were measured as described above.

#### 2.5.2. Drug-Induced Rotation Asymmetry

Drug-induced rotation asymmetry was recorded manually for 5 min following ketamine injection (10 mg/kg intramuscular) [28,29] in a plastic cylinder with a smooth round bottom (30 cm bottom diameter and 40 cm walls height). Intramuscular injection of ketamine in subanesthetic dose to the rats with unilateral occlusion of the middle cerebral artery causes an increase in ipsilateral (toward the injured hemisphere) rotation. A full 360° arc of turning in the same direction scored as one rotation. The percentage of rotations to the right (ipsilateral to the injured hemisphere) was assessed relative to the total number of rotations in both directions.

### 2.6. Brain Infarct Analysis

After 21 days of testing, the animals were sacrificed by CO_2_ inhalation, and their brains were perfusion-fixed. The heart was exposed by a median sternotomy, and the ascending aorta was catheterized via the left ventricle. The blood was removed from animals with 150 mL saline solution containing heparin (100 Units/L) perfusion at a constant perfusion rate of 30 mL/min. Then the perfusion medium was switched to a mixture of 3% (*w*/*v*) formaldehyde buffered solution (pH 7.4) and 1% (*v*/*v*) glutaraldehyde solution (150 mL). Following brain perfusion-fixation, the animals were decapitated, and the brain was removed from the skull. The brain was cryoprotected in sucrose buffered solutions (pH 7.4) of graded concentrations (10%, 20%, 30%) and then immediately frozen in the cryostat (Microm HM 525, Carl Zeiss) at −65°C. From each brain, coronal cryosections of 50 μm thickness at 1 mm intervals were obtained and stained with cresyl violet. The stained sections were scanned and processed with the 3D-reconstruction program ‘Reconstruct’ https://synapseweb.clm.utexas.edu/software-0 (accessed on 24 August 2021). Volumes of both hemispheres were calculated using the Cavalieri method [30]. The indirect lesion area was calculated as the intact area of the ipsilateral hemisphere subtracted from the area of the contralateral (uninjured) hemisphere. Lesion volume was calculated as a percentage in which the lesion volume was compared with the contralateral hemisphere volume [31].

### 2.7. Statistical Analysis

The behavioral data and infarct size were analyzed using Statistica for Windows 7.0. Test for the normality was performed for all data. All the values are presented as mean ± standard deviation. The significance of the differences in the rotarod test and ketamine-induced rotation test was evaluated by repeated measures ANOVA with post hoc Duncan analysis. The significance of the differences when comparing the volumes of cerebral infarction was assessed using one-way ANOVA.

## 3. Results

### 3.1. Behavioral Testing

#### 3.1.1. Motor Performance after MCAO in Rotarod Test

**Figure 1:**
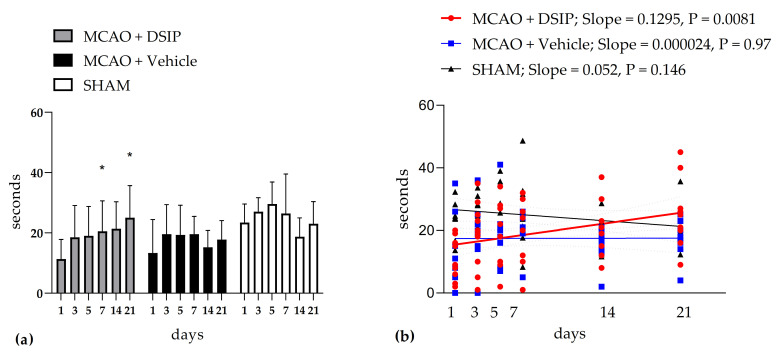
Latent time of falling from a rotating rod: (**a**) Results are presented as group mean ± standard deviation. * *p* < 0.01 relative to the first day of testing according to repeated measures ANOVA, Duncan’s test. (**b**) Linear regression was used to assess the dynamic changes in the latency time of falling. The individual values are given for all groups for each day of testing. The slopes of the linear regression lines for each group are shown. The slope of the regression line for the DSIP-treated group was significantly different from 0, indicating that the time of fall is dependent on the day of testing. Thus, there was a dynamic improvement in the performance of the rotarod test over 21 days of testing in DSIP-treated animals.

Animals treated with DSIP showed a significantly improved performance in a rotarod test (Figure 1). Motor coordination recovered on the 7-th day of testing, which was confirmed by the repeated measures ANOVA analysis. Linear regression analysis was applied to assess the dynamics of the fall latency. Only in animals receiving DSIP were there positive dynamics of this parameter, which was confirmed by statistical assessment of the slope of the linear regression line (Figure 1). These findings elucidated a positive DSIP effect on the recovery of motor performance after stroke. Individual data for all animals are given in the Appendix A.

#### 3.1.2. Ketamine-Induced Rotation

**Figure 2:**
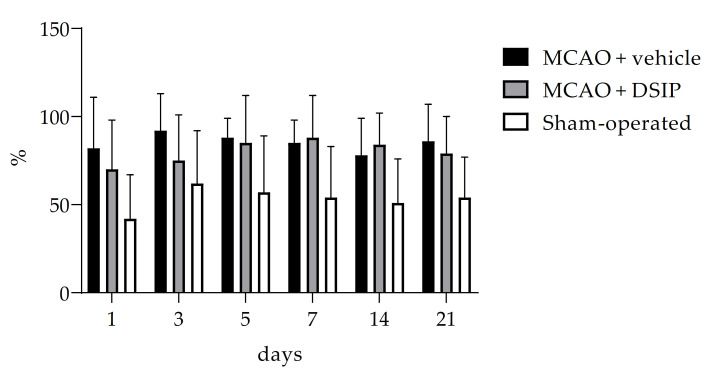
The percentage of the animal’s rotation in the direction of damage relative to rotations in both directions. Results are presented as group mean + standard deviation.

As can be seen from Figure 2, there was no significant influence of DSIP on rotation asymmetry tested by the ketamine-induced rotation test. Individual data for all animals are given in the Appendix A.

#### 3.1.3. Brain Infarction Volume

**Figure 3:**
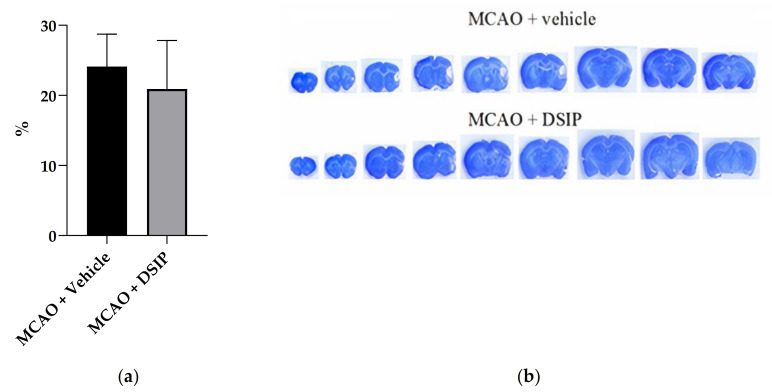
Brain damage: (**a**): The percentage of damage to the right hemisphere. Results are presented as group mean ± standard deviation. (**b**) Representative slices of MCAO + vehicle animal and MCAO + DSIP animal. Cryoslices stained with cresil violet, 50 µm thick.

The brain infarction volume in the group MCAO+DSIP was 20.9 ± 6.9% versus 24.1 ± 4.6% in the group MCAO + vehicle, which had no statistical significance (Figure 3). Individual data for all animals are given in the Appendix A.

## 4. Discussion and Conclusions

Although the infarct volume of the affected hemisphere in DSIP-treated animals was less than in vehicle-treated animals, this difference was not significant. In addition, there was not much difference in the ketamine-induced rotation between DSIP-treated animals and vehicle-treated animals. However, in the test of motor performance, the latent time of falling from the rotarod significantly increased only in DSIP-treated animals. This increase was confirmed by both linear regression analysis and repeated measures ANOVA. Motor dysfunction is one of the most serious complications of stroke [32]. Loss of motor ability is thought to be caused by abnormalities in the neural network that controls movement [33,34,35,36,37]. Motor recovery is crucial for regaining independence [38]. Rehabilitation of stroke survivors includes maximal recovery of motor functions and coordination [39,40,41]. Using different biomarkers to determine the motor functions of patients is helpful in the prediction of motor recovery [42,43,44,45,46,47]. We assessed motor function dynamics for stroke-subjected animals. The obtained results allow us to reveal a rather significant positive DSIP effect on the recovery of motor coordination, which is an important parameter in stroke-subjected animals. This DSIP effect could be associated with the rescuing of neurons of the motor cortex and subcortical structures associated with motor control. Neurons rescuing mechanisms may include DSIP effects on biosynthetic processes in the brain, as has been previously reported [48]. It is suggested that the protective effects of peptide could be based on a decline in the synthesis rate of stress protein factors [49]. The motor function recovery of animals treated with DSIP may also be associated with its effect on glutamate receptors and GABA receptors of neurons of the cortex, thalamus, hypothalamus, hippocampus, and cerebellum. Thus, Sudakov and colleagues showed that DSIP-caused activation of neurons at the sensorimotor cortex, dorsal hippocampus, ventral thalamic nuclei, and lateral hypothalamus could be explained by its action on NMDA receptors of these structures [50]. Grigor’ev et al. showed that DSIP potentiated GABA-activated currents in hippocampal and cerebellar neurons and blocked NMDA-activated potentiation in cortical and hippocampal neurons [51]. There are available data that DSIP improves mitochondrial respiratory activity. Oxygen utilization by mitochondria is carried out through the respiratory chain—a system of specific peptides involved in electron transport and ATP synthesis. The combination of these processes ensures the normal functioning of the enzymatic energy system. Khvatova et al. showed that pretreatment of rats with DSIP prior to their subjection to hypoxia completely inhibited the hypoxia-induced reduction of mitochondrial respiratory activity [52].

It is known that in the process of ischemia, a cascade of metabolic reactions is activated, some of which are involved in the production of reactive oxygen species (ROS) and reactive nitrogen species (RNS), such as superoxide, hydrogen peroxide [H_2_O_2_], hydroxyl radical, nitric oxide [NO], and peroxynitrite, which cause most of the damage observed in ischemia-reperfusion [53,54,55,56]. The antioxidant mechanism of DSIP is an increase in the activity of superoxide dismutase (SOD) and glutamate peroxidase (GPO) [56], mediated by an increase in the expression of genes encoding these enzymes, as shown in aging animals [57,58].

In our other study [59], it was shown that the administration of DSIP and its analog KND-peptide during occlusion led to the death of animals, and when administered during the reperfusion, a decrease in the size of the brain and heart infarction was observed. Presumably, mortality could be a consequence of the inactivation of the genes for the early response of c-Fos upon administration of DSIP during occlusion, whose activity is associated with the activation of NMDA receptors [60,61]. In the present study, a combined administration of DSIP was used preventively (before the onset of occlusion) and then within 7 days after reperfusion. Thus, we managed to avoid the negative consequences of the introduction of DSIP during the period of occlusion and, on the contrary, to demonstrate a positive effect on the restoration of motor functions. In our study, we demonstrated that DSIP might have clinical benefits for stroke therapy in terms of accelerating the recovery of motor functions after a stroke. Research into the DSIP effect on stroke should be focused on in future studies of optimal dosage regimens to provide more effective and fast recovery from stroke.

## Data Availability

All raw data are presented in Appendix A.

## Appendix A

### Appendix A.1. Raw Data on Rotarod

**Table A1:** Latent time of falling from rotarod for SHAM-operated animals, sec.

Animal Number	1	2	3	4	5	6	7	Mean ± SD
Day 1	14	25	32	17	24	28	24	23 ± 6
Day 3	28	21	31	25	22	28	34	27 ± 5
Day 5	21	26	39	36	21	35	29	30 ± 7
Day 7	21	8	49	18	32	33	24	26 ± 13
Day 14	12	12	29	22	16	17	23	19 ± 6
Day 21	23	25	12	19	20	26	36	23 ± 7

**Table A2:** Latent time of falling from rotarod for vehicle-treated animals, sec.

Animal Number	8	9	10	11	12	13	14	15	16	Mean ± SD
Day 1	5	11	15	26	5	15	32	0	8	13 ± 11
Day 3	20	15	19	36	14	25	25	0	22	20 ± 10
Day 5	16	8	21	22	20	21	41	7	18	19 ± 10
Day 7	20	19	19	26	21	24	21	5	21	20 ± 6
Day 14	16	17	14	20	21	18	16	2	13	15 ± 5
Day 21	23	14	17	18	23	16	25	4	20	18 ± 6

**Table A3:** Latent time of falling from rotarod for DSIP-treated animals, sec.

Animal Number	17	18	19	20	21	22	23	24	25	26	Mean ± SD
Day 1	15	8	9	2	16	6	3	19	20	15	11 ± 6
Day 3	18	25	10	1	19	5	35	20	23	29	19 ± 11
Day 5	18	27	10	2	18	9	22	28	22	34	19 ± 10
Day 7	24	30	10	1	20	12	20	30	26	32	20 ± 10
Day 14	30	37	12	8	23	19	21	19	15	30	21 ± 9
Day 21	45	21	16	9	25	20	21	27	26	40	25 ± 11

### Appendix A.2. Raw Data on Ketamine-Induced Rotation

**Table A4:** The percentage of the animal’s rotation in the direction of damage relative to rotations in both directions for SHAM-operated animals, %.

Animal Number	1	2	3	4	5	6	7	Mean ± SD
Day 1	58	26	8	32	52	33	83	42 ± 25
Day 3	72	64	18	25	55	100	100	62 ± 30
Day 5	92	59	0	30	67	59	93	57 ± 32
Day 7	22	61	16	92	52	65	71	54 ± 29
Day 14	49	52	4	56	62	48	85	51 ± 25
Day 21	64	41	19	58	37	64	92	54 ± 23

**Table A5:** The percentage of the animal’s rotation in the direction of damage relative to rotations in both directions for vehicle-treated animals, %.

Animal Number	8	9	10	11	12	13	14	15	16	Mean ± SD
Day 1	94	39	82	100	25	100	100	100	96	82 ± 31
Day 3	90	93	92	90	75	100	100	100	85	92 ± 24
Day 5	76	76	100	100	100	88	93	61	100	88 ± 13
Day 7	96	96	85	93	77	73	65	89	95	85 ± 13
Day 14	70	100	93	85	90	95	9	91	67	78 ± 22
Day 21	72	85	83	76	83	86	100	98	92	86 ± 21

**Table A6:** The percentage of the animal’s rotation in the direction of damage relative to rotations in both directions for DSIP-treated animals, %.

Animal Number	17	18	19	20	21	22	23	24	25	26	Mean ± SD
Day 1	71	59	0	69	67	71	100	75	100	87	70 ± 32
Day 3	73	90	64	100	41	67	100	33	100	80	85 ± 29
Day 5	100	100	81	100	0	100	100	79	95	91	85 ± 29
Day 7	83	100	81	100	100	100	77	50	92	95	88 ± 24
Day 14	83	100	38	99	91	95	100	67	64	98	84 ± 18
Day 21	84	100	53	100	92	79	95	40	55	94	79 ± 22

### Appendix A.3. Raw Data on Brain Damage

**Table A7:** The percentage of damage to the right hemisphere for vehicle-operated animals, %.

Animal Number	8	9	10	11	12	13	14	15	16	Mean ± SD
	21.0	25.9	26.2	19.1	26.8	23.5	22.0	22.6	18.9	24.1 ± 4.6

**Table A8:** The percentage of damage to the right hemisphere for DSIP-treated animals, %.

Animal Number	17	18	19	20	21	22	23	24	25	26	Mean ± SD
	18.0	20.4	18.9	26.2	15.5	24.2	31.0	6.8	20.6	27.8	20.9 ± 6.9
